# Evaluation of a communication skills seminar for students in a Japanese medical school: a non-randomized controlled study

**DOI:** 10.1186/1472-6920-4-24

**Published:** 2004-11-18

**Authors:** Kei Mukohara, Kazuya Kitamura, Hideki Wakabayashi, Keiko Abe, Juichi Sato, Nobutaro Ban

**Affiliations:** 1 Department of General Medicine, Nagoya University Graduate School of Medicine 65 Tsurumai-cho Showa-ku, Nagoya, 466-8560, Japan

## Abstract

**Background:**

Little data exist for the effectiveness of communication skills teaching for medical students in non-English speaking countries. We conducted a non-randomized controlled study to examine if a short intensive seminar for Japanese medical students had any impact on communication skills with patients.

**Methods:**

Throughout the academic year 2001–2002, a total of 105 fifth-year students (18 groups of 5 to 7 students) participated, one group at a time, in a two-day, small group seminar on medical interviewing. Half way through the year, a five-station objective structured clinical examination (OSCE) was conducted for all fifth-year students. We videotaped all the students' interaction with a standardized patient in one OSCE station that was focused on communication skills. Two independent observers rated the videotapes of 50 students who had attended the seminar and 47 who had not. Sixteen core communication skills were measured. Disagreements between raters were resolved by a third observer's rating.

**Results:**

There was a statistically significant difference in proportions of students who were judged as 'acceptable' in one particular skill related to understanding patient's perspectives: asking how the illness or problems affected the patient's life, (53% in the experimental group and 30% in the control group, p = .02). No differences were observed in the other 15 core communication skills, although there was a trend for improvement in the skill for asking the patient's ideas about the illness or problems (60% vs. 40%, p = .054) and one of the relationship building skills; being attentive and empathic nonverbally (87% vs. 72%, p = .064).

**Conclusion:**

The results of this study suggest that a short, intensive small group seminar for Japanese medical students may have had a short-term impact on specific communication skills, pertaining to understanding patient's perspectives.

## Background

The literature from English-speaking countries indicates that teaching communication skills is effective in improving learners' communication skills with patients [1]. However, the evidence from non-English speaking countries is sparse [1]. In addition, the conceptual frameworks for communication skills teaching are based on research evidence from English-speaking countries [2]. There is an ongoing debate about whether the principles and methods for teaching communication skills developed in English-speaking countries could be applied to other places with different languages and cultures [2-4].

Teaching communication skills is gaining popularity and proliferating for Japanese health professional students [5]. Yoshida et al. conducted a controlled study to examine the effects of such training with 16 Japanese dental students and had a positive result [6]. A few reports have been published on Japanese medical students [7-9]. However, to our best knowledge, no controlled studies for communication skills teaching have been conducted for that population.

In many traditional medical schools in Japan, communication skills teaching is limited in time and scope, and isolated from other formal curricula. Thus it is important to know whether such type of training make a difference, at least in the short run. This should also be of interest to educators elsewhere who similarly work in settings where there is not enough formal curricular time for communication skills teaching.

The objective of this study was to evaluate the impact of a short, intensive small group seminar, which was based on Western educational principles, on Japanese medical students' communication skills with patients.

## Methods

### Participants

Medical schools in Japan last six years with the last two years consisting of clerkships. Before the fifth-year, Japanese students typically have few direct interactions with patients. Throughout the academic year 2001–2002, a total of 105 fifth-year students from the Nagoya University School of Medicine rotated through the various clinical services of the Nagoya University Hospital. Students divided themselves into 18 groups of 5 to 7, but the sequential order of rotations is set by the medical school officials.

### Educational intervention

**Figure 1:**
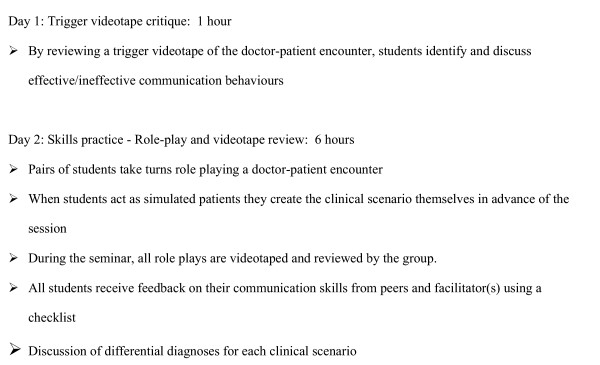
Learning activities during a two-day seminar on medical interviewing and communication skills.

As part of a 1-week clerkship rotation at the Department of General Medicine, students participated in a two-day, small group seminar on the medical interview and communication skills. Typically either or both of two of the authors (KM, NB) facilitated the seminar. Both facilitators had had an experience in learning and teaching the medical interview and communication skills in the United States. The seminar utilized learner-centered, skills-oriented, experiential, and interactive learning methods. To guide the teaching of communication skills, we created a conceptual model for patient-physician communication referring to 3 existing models [2,10,11]. Although our main teaching focus is on communication process skills, we also addressed the content aspects of the medical interview (e.g., discussion of differential diagnosis). The learning activities during the seminar are summarized in Figure 1.

### Outcome measures

In September 2001, half way through the academic year, a five-station objective structured clinical examination (OSCE) was conducted for all fifth-year students. The primary purpose of the OSCE was to provide trainees with the opportunity to receive feedback on their clinical skills from the faculty in a safe and structured environment. One OSCE station focused on the medical interview. Students engaged in a 5-minute interaction with a standardized patient presenting with cough. A total of 10 fourth-year students were trained to serve as standardized patients in a series of 3 small group sessions, each lasting 60 minutes [12]. During the interview, the fifth-year students were observed by faculty and evaluated for both station-specific and general communication skills on the pre-defined rating scale. The faculty gave students a 3-minute feedback immediately after the encounter. Standardized patients did not give feedback. All interactions were videotaped and subsequently reviewed by faculty members to provide students with additional written feedback.

Placed at the mid point of the academic year, the OSCE provided us with the opportunity to evaluate the short-term effectiveness of the small group seminar on students' communication skills. We reviewed the videotapes of 52 students who had attended the seminar (the experimental group) and 53 students who had not at the time of the OSCE (the control group). The group assignment was based on the sequential order of clinical rotations, arbitrarily set by the medical school officials. The time intervals between the seminar and the OSCE ranged from 1 week to 5 months. Students were asked to provide informed consent using a form that had been approved by the Institutional Review Board at the Nagoya University Hospital.

The interview rating form was created by one of the authors (KM) and includes 16 essential communication skills items. They are grouped into 6 communication tasks that should be accomplished during the initial 5 minutes of an encounter (*establish initial rapport, survey patient's reason(s) for the visit, determine the patient's chief concern, elicit and understand the patient's perspective, manage flow – provide the structure for the interview, and use of relationship building skills*). The performance was rated on a 4-point scale labelled as good, satisfactory, insufficient and poor. The skills items were selected for their association with improved patient outcomes. They were derived from evidence-based communication assessment tools (i.e., *the Calgary-Cambridge observation guide, the SEGUE framework, and the checklist developed by the investigators of the Macy Initiative in Health Communication*) [2,10,11]. These instruments are based on the same conceptual models for patient-physician communication we referred to during our teaching seminar.

Two staff members (KK, HW) were trained to serve as raters. The tapes were independently reviewed and scored using the students' communication skills rating scale. Ten arbitrarily selected videotapes of students' role-plays of a doctor-patient encounter during the small group seminar were used to ensure accuracy and inter-rater reliability. At the time of the research the raters primarily worked outside the University and did not participate in the teaching seminars. Thus they were blinded to the students' group assignments.

From the 105 students who attended the OSCE, 2 did not return the consent form, 5 did not give permission for the video review, for 1 the videotape quality was too poor to be analyzed. Thus, a total of 97 videotapes were available for the analysis.

A skill item was considered 'acceptable' if both raters scored the students' performance as 'good' or 'satisfactory.' It was labeled as 'unacceptable' if both raters scored the performance as 'insufficient' or 'poor.' When these two raters disagreed over the judgment about the students' performance (e.g., one rater scored the performance of a skill item as 'acceptable' and the other scored the performance of the same item as 'unacceptable'), a communication educator and researcher (KA) served as the tiebreaker. The overall disagreement rate between the two raters (KK, HW) was 21%. The raters disagreed more often on inviting the patient to tell the story chronologically (41%), actively responding to the patient's concerns and nonverbal cues (34%), and being attentive and empathic nonverbally (31%).

### Statistical analysis

We compared baseline characteristics of the two groups using t-tests for a continuous variable (age) and chi-square tests for categorical variables. To evaluate the effect of the educational intervention, the proportion of students with 'acceptable' performance was compared with those whose performance was unacceptable using chi-square tests for all 16 skills items. All statistical analyses were done by JS using Stat View Version 5.0 (SAS Institute Inc. North Carolina).

## Results

Student characteristics including gender did not differ between the groups except that more students in the control group engaged in self study as a preparation for the OSCE (p < .05) (Table 1). There were trends that more students in the control group took an elective on communication skills at the 4^th ^year and were interested in a future generalist career. For both groups combined, the mean age was 23.5 years and 38 % were women.

**Table 1:** Baseline characteristics of the students

	Intervention Group (N = 47)	Control Group (N = 50)	P-value
Mean age (SD)	23.6 (1.5)	23.4 (1.5)	0.48
Women	36% (N = 17)	40% (N = 20)	0.70
Did a self-study preparing for OSCE	34% (N = 16)	56% (N = 28)	0.03
Took an elective on communication in medicine at the 4^th ^year	43% (N = 20)	54% (N = 27)	0.26
Interested in becoming a generalist	13% (N = 6)	26% (N = 13)	0.10

The proportions of students who were judged to have performed as 'acceptable' for each of the 16 items are shown in Table 2. There was a statistically significant difference for one particular skill related to understanding patient's perspectives: "exploring how the illness or problem affected the patient's life" (53% in the intervention group vs. 30% in the control group, p = .02). No significant differences were observed for the other 15 skills, although there was a trend favouring the intervention in the skill for "asking the patient about ideas concerning the illness or problem (60% vs. 40%, p = .054) and one of the relationship building skills: "being attentive and empathic nonverbally (87% vs. 72%, p = .064)."

**Table 2:** Student performance of the skill judged as 'acceptable'

Communication Tasks and Related Skills	Intervention Group (N = 47)	Control Group (N = 50)	P-value
*Establish Initial Rapport*			
Greet patient and obtain patient's name	92%	94%	0.43
Introduce self and clarify the role	100%	98%	1.0
*Survey Patient's Reason(s) for the Visit*			
Allow the patient to complete his/her opening statement	9%	6%	0.71
Invite the patient to tell the story chronologically	49%	46%	0.77
Actively listen, using verbal and nonverbal techniques	66%	58%	0.42
Summarize. Check for understanding. Invite more questions?	70%	60%	0.29
*Determine the Patient's Chief Concern*			
Ask closed-questions that are non-leading, one at a time	100%	100%	1.0
Define the concern completely	96%	94%	1.0
*Elicit and Understand the Patient's Perspective*			
Explore contextual factors (e.g., job, family, hobbies)	66 %	62%	0.69
Ask the patient's ideas about the illness or problems	60%	40%	0.054
Explore how the problem affects the patient's life	53%	30.0%	0.02
*Manage Flow – Provide the Structure to the Interview*			
Summarize periodically throughout the interview	81%	76%	0.56
Use signposting	40%	30%	0.28
*Use of Relationship Building Skills*			
Be attentive and empathic nonverbally	87%	72%	0.064
Actively respond to patient's concerns and nonverbal cues	38%	40%	0.8637
Use appropriate language	100%	100%	1.0

## Discussion

A short, intensive small group seminar on medical interviewing appeared to have had an impact on some specific skills, pertaining to "eliciting and understanding the patient's perspectives." It did not seemed to have improved the skills associated with the other tasks: establishing initial rapport, surveying the patient's reason(s) for the visit, determining the patient's chief concern, and managing flow – providing the structure for the interview, and the skills for building relationships.

There are several strengths of our study. First, this is one of the few empirical, controlled studies from a non-English speaking country. Even though the students were not strictly randomized into intervention and control groups, the assignment occurred arbitrarily by the administration, without regard to students' preferences or interests in medical interviewing. Thus, it is unlikely that the higher scores in the intervention group are attributable to self-selection. Although there was a significant difference between the groups in proportions of students who did a self-study for the OSCE, which might have caused the results of no difference in most of the skills, the other characteristics such as age and gender were similarly distributed (Table 1). Second, interventions and evaluations were guided by the conceptual framework, modelled after the 3 widely used theoretical models that are based on rigorous, empirical research in the field of patient-physician communication [2,10,11]. Third, the communication skills evaluation instrument was matched with the competencies taught in the small group sessions [13]. By carefully delineating and defining specific communication skills that should be addressed in the teaching session and by evaluating the effect of the teaching intervention on these individual skills, we sought to examine whether some skills were more teachable than others in such a brief, small group sessions.

Our study also has weaknesses that should be addressed. First, our teaching method was based on the research findings in Western world, and this is based on the untested assumption that these findings are equally valid in Japan. There is evidence that patient-physician communication patterns in Japan are different from those in the West. Previous research by Ohtaki and colleagues compared patient-physician communication patterns in Japan and the USA [14]. It included 20 outpatient consultations of four physicians in Japan and 20 outpatient consultations of five physicians in the USA. Japanese physicians spent less time on social talk than the USA counterparts (5% vs. 12%). Japanese patient-physician encounters included more pauses than those in the USA (30% vs. 8.2% of the total consultation length). There is a need for more empirical studies linking physicians' communication skills to patient outcomes specifically for Japanese population. Second, our assessment of students' communication skills was based on observations of a single, five-minute OSCE station. The reliability of which as a measure of communication skills is known to be low [15]. Third, because we assessed the students' skills at only one time, we could not assess the change in students' performance before and after the intervention. Fourth, the use of junior students as standardized patients may have influenced the performance of the examinees. The accuracy of student-standardized-patients' (student-SPs') portrayal would be a critical issue especially when the OSCE is used to grade students. Although we did not objectively investigate the consistencies of the portrayal by student-SPs, our examinees rated highly the fidelity of student-SPs, i.e., the degree to which they were acting as if they were real patients (mean score, 3.9 on a 5-point Likert scale) [12]. Fifth, our study might have only shown that the intervention was effective in improving students' skills for eliciting 'expert' observations of patient perspectives, not actual patient perspectives. We did not ask student-SPs whether examinees elicited their perspectives. Rather, we judged examinees' ability to elicit patient perspectives through their 'observable' behaviours from the experts' point of view. The role of student-SPs in evaluating fellow students' communication skills, particularly skills for eliciting patient perspectives should be addressed in future studies. Finally, the statistically significant difference observed for only 1 skill among a total of 16 skills could be due to chance alone. It is certainly possible that our intervention was too weak to influence any of the 16 communication skills.

One can hypothesize the reasons why the intervention appeared to make a difference to some communication skills competencies but not to others. One could speculate that the competencies that were not influenced by the intervention were either very easy in general or too difficult to acquire in such a short teaching session. For example, the skills for establishing initial rapport (greet patient and obtain the patient's name, introduce self and clarify roles) and skills for determining the patient's chief concern (ask closed-ended questions that are non-leading and one at a time, define the concern completely) may be already present from the outset or so easy to acquire that a self-study just before the OSCE would make no differences in scores between the groups regardless of the intervention. On the other hand, the skills for surveying the patient's reason(s) for the visit, which requires being open at the beginning of the interview, may be too difficult for students to demonstrate, with or without the intervention. In particular, only 9% in the intervention group and 6% in the control group demonstrated an acceptable performance for the skills of allowing patients to complete their opening statements. These very low scores may also indicate that during small group sessions, we did not emphasize enough the importance of not interrupting patients at the beginning of the interview. Another explanation is that 'content' skills (i.e., what we communicate) are easier for students to acquire than 'process' skills (i.e., how we communicate). Kurtz at al. noted that the skills for understanding patient's perspectives, which our intervention made a difference, are actually 'content' skills, not 'process' skills [16]. One could argue that the intervention was just too short to influence other 'process' skills. These interesting hypotheses should require further investigations.

## Conclusions

The results of this study suggest that a short, intensive small group seminar for Japanese medical students may have had an impact on specific communication skills, namely, skills for exploring how the illness or problem affected the patient's life, asking the patient about ideas concerning the illness or problem, and being attentive and empathic nonverbally at least in the short term. Further studies should be done to confirm this preliminary finding and to clarify the skills for which educational interventions could make a difference.

## Competing interests

The author(s) declare that they have no competing interests.

## Authors' contributions

KM contributed to the conception and design of the study, design and implementation of the educational intervention, interpretation of the data, and drafting of the manuscript.

KK and KW contributed to the collection of the data and reviewing of the manuscript.

KA contributed to the collection of the data and reviewing of the manuscript.

JS contributed to the conception and design of the study, analysis and interpretation of the data and reviewing of the manuscript.

NB contributed to the conception and design of the study, design and implementation of the educational intervention, interpretation of the data, and reviewing of the manuscript.

## Pre-publication history

The pre-publication history for this paper can be accessed here:


